# P-1389. Predictors for False Positive Interferon Gamma Release Assays in the San Antonio Market of the Military Health System

**DOI:** 10.1093/ofid/ofaf695.1576

**Published:** 2026-01-11

**Authors:** John Kim, Joseph Marcus, Diane Homeyer

**Affiliations:** Brooke Army Medical Center, San Antonio, TX; Brooke Army Medical Center, San Antonio, TX; Brooke Army Medical Center, San Antonio, TX

## Abstract

**Background:**

Identification of patients with latent tuberculosis infection (LTBI) in high-risk populations is critical in global efforts to eradicate tuberculosis. However, false positive rates with interferon gamma release assays (IGRAs) can be high when screening low-risk populations. Factors associated with false-positive QuantiFERON-TB Gold Plus (QFT-Plus) testing have not been described in the United States military.

**Methods:**

All patients with a positive QFT-Plus assay between January 2022 and June 2024 at Joint Base San Antonio were included for further analysis. Patients were deemed to have true infection (either active or latent disease) or a false positive QFT-Plus test based on determination by the treatment team. Demographic and clinical information was compared between true positive and false positive groups using the Fisher’s exact test for nominal variables and Mann-Whitney U test for continuous variables.

**Results:**

A total of 192 patients were included with 99 (52%) true positive results, 12 (6%) false positive, 60 (31%) previously treated patients, and 21 (11%) with incomplete follow-up (Table 1). Most patients who tested positive were serving in the military (53%) and the most common indication for testing was occupational screening. Twenty-three (23%) patients with true positives had no risk factors for tuberculosis. Comparing those with latent or active tuberculosis to those with a false positive test, there were no significant differences by demographics, risk factors, ordering location, or indication for testing (Table 2). However, the TB – nil was significantly greater in true positive patients (0.968 [0.52-3.52] vs. 0.435 [0.38-0.62], p = 0.00072).

**Conclusion:**

Despite the need for targeting high-risk populations, most IGRAs in this large military market are ordered to screen low risk patients. There was no difference in demographic or clinical risk factors between those with true positives and false positives, but there were quantitative differences in the results of the IGRA.

**Disclosures:**

All Authors: No reported disclosures

